# A Practical Treatise on Diseases of the Hair and Scalp

**Published:** 1887-10

**Authors:** 


### BOOK NOTICES.

A Practical Treatise on the Diseases of the Hair and Scalp. By Geo. Thomas Jackson, M. D. Pp. 356; price, $2.75. New York: E. B. Treat. 1887.

Dr. Jackson's book is a timely one, as the subject treated in it has never been so well and so thoroughly covered.

The first part of the volume is devoted to the anatomy and physiology of the hair; subsequent chapters to the many diseases of the hair and scalp. So far as the clinical history and the pathology of these diseases are given the book will be of value to homœopaths, but the treatment recommended is chiefly local, and there we can do much better with our internal medication. We can commend the volume to all who would possess a complete medical library.

